# Satellite imagery and machine learning for identification of aridity risk in central Java Indonesia

**DOI:** 10.7717/peerj-cs.415

**Published:** 2021-05-18

**Authors:** Sri Yulianto Joko Prasetyo, Kristoko Dwi Hartomo, Mila Chrismawati Paseleng

**Affiliations:** Faculty of Information Technology, Satya Wacana Christian University, Salatiga, Central Java, Indonesia

**Keywords:** Vegetation indices, Machine learning, Remote sensing, Aridity

## Abstract

This study aims to develop a software framework for predicting aridity using vegetation indices (*VI*) from LANDSAT 8 OLI images. *VI* data are predicted using machine learning (*ml*): Random Forest (RF) and Correlation and Regression Trees (CART). Comparison of prediction using Artificial Neural Network (ANN), Support Vector Machine (SVM), k-nearest neighbors (k-nn) and Multivariate Adaptive Regression Spline (MARS). Prediction results are interpolated using Inverse Distance Weight (IDW). This study was conducted in stages: (1) Image preprocessing; (2) calculating numerical data extracted from the LANDSAT band imagery using vegetation indices; (3) analyzing correlation coefficients between *VI*; (4) prediction using RF and CART; (5) comparing performances between RF and CART using ANN, SVM, k-nn, and MARS; (6) testing the accuracy of prediction using Mean Square Error (MSE) and Mean Absolute Percentage Error (MAPE); (7) interpolating with IDW. Correlation coefficient of *VI* data shows a positive correlation, the lowest r (0.07) and the highest r (0.98). The experiments show that the RF and CART algorithms have efficiency and effectivity in determining the aridity areas better than the ANN, SVM, k-nn, and MARS algorithm. RF has a difference between the predicted results and 1.04% survey data MAPE and the smallest value close to zero is 0.05 MSE. CART has a difference between the predicted results and 1.05% survey data MAPE and the smallest value approaching to zero which is 0.05 MSE. The prediction results of *VI* show that in 2020 most of the study areas were low vegetation areas with the Normalized Difference Vegetation Index (NDVI) < 0.21, had an indication of drought with the Vegetation Health Index (VHI) < 31.10, had a Vegetation Condition Index (VCI) in some areas between 35%–50% (moderate drought) and < 35% (high drought). The Burn Area Index (dBAI) values are between −3, 971 and −2,376 that show the areas have a low fire risk, and index values are between −0, 208 and −0,412 that show the areas are starting vegetation growth. The result of this study shows that the machine learning algorithms is an accurate and stable algorithm in predicting the risks of drought and land fire based on the VI data extracted from the LANDSAT 8 OLL imagery. The VI data contain the record of vegetation condition and its environment, including humidity, temperatures, and the environmental vegetation health.

## Introduction

Based on the study of disaster risk in Central Java Province, Indonesia, the number of people potentially exposed to drought disasters was estimated at 33.7 million people. Economic losses as a result of exposure to the drought were estimated at 65.2 billion rupiahs, and damage to the land area of 61 thousand hectares (Deputi Bidang Pencegahan dan Kesiapsiagaan, 2016). Drought is a disaster that occurs with a slow onset and can spread in wide areas, so that it has an impact on food security, the economy and social security (Çelik & Gülersoy, 2018). In most countries in the world, including Indonesia, drought is determined based on the rainfall data collected from meteorological stations (Nam et al., 2018). The method commonly used to calculate the amount of drought index is the Standardized Precipitation Index (SPI) method. The drought index is calculated by comparing the magnitude of deviations in the amount of rainfall from normal conditions or the long-term average in a particular region (Bayissa et al., 2017). Drought assessment using the SPI method and meteorological data has high accuracy and precision, especially in areas with uniform density and distribution of meteorological stations. However, in developing countries, the density and distribution of meteorological stations and limited operational costs make it difficult to obtain rainfall measurement data. The limitation of rainfall data becomes an obstacle in identifying the intensity, frequency and spatial distribution of drought (Dutta et al., 2015a). The SPI method is one of the 150 methods developed as drought indices. Apart from the SPI, the other common methods used in drought studies are the Standardized Precipitation Evapotranspiration Index (SPEI), the Palmer Drought Severity Index (PDSI), and the Precipitation Anomaly Percentage (Wang et al., 2020). The SPEI is the index used to take into account the drought damage based on the climate balance between precipitation and evapotranspiration. The timescale of SPEI ranges from 1-24 months and is interpolated using high resolutions in order to obtain vegetation responses toward potential water deficit over different timescales (Gouveia et al., 2017). The Palmer Drought Severity Index (PDSI) is an estimate of relative dryness in an area using temperature, precipitation, and soil characteristics. In general, the PDSI used to take into account of long-term drought which has affected an area for more several months (Mika et al., 2005). The PDSI works based on the water supply and water demand system while considering: (1) potential evapotranspiration calculated using Thornwaite method, (2) the amount of measured soil moisture, (3) the reduced amount of soil moisture due to evapotranspiration, (4) runoff. The Precipitation Anomaly Percentage (PAP) is precipitation anomaly percentage index which reflects hydrology factors deviating from the same average periods over years. The PAP index shows drought degrees on the same periods (Zhengmin et al., 2014).

In the late 20^th^ or early 21^st^ century, satellite imagery was widely used to monitor the risk of drought, both spatially and temporally. Satellite imagery data was chosen because it was available with high spatial and temporal resolution and can be obtained free of charge. The satellite imagery data will be extracted to get numerical data of vegetation indices, which are the main data to classify environmental conditions such as drought, moisture stress and temperature on the earth’s surface. Vegetation indices are numerical values of the results of spectral transformation of image-forming bands that are formulated to assess the photosynthetic activity of the vegetation canopy layer which correlates to plant types, water content in tissues, and photosynthetic activities. Vegetation Indices are mathematical combination of different spectral bands, and are designed to visualize various features of different images (Karakacan Kuzucu & Bektas Balcik, 2017). The vegetation index is dimensionless radiometric measurements acquired from linear combination of the red spectrum and NIR, which is calculated based on the light reflectance from satellites, or light reflectance from various objects on the earth surface (Gherghina, Maftei & Filip, 2011). Various aridity indices have been formulated by experts for classifying drought, including: (1) index for monitoring vegetation conditions as a result of drought (Vegetation Condition Index or VCI), (2) index for monitoring the surface temperature of the earth (Temperature Condition Index or TCI), (3) index for monitoring soil moisture (Microwave Integrated Drought Index or MIDI), (4) index for monitoring the combination of temperature and humidity on the earth’s surface and their impact on vegetation health (Vegetation Health Index or VHI) and (5) index for monitoring the level of vegetation greenness (Normalized Difference Vegetation Index or NDVI) (Elhag & Zhang, 2018). The most suitable method used for the purpose of representation, categorization and classification of vegetation indices is machine learning (Maxwell, Warner & Fang, 2018). In essence, the machine learning algorithm shows how a machine compiles and learns to create valid information from observational and historical data. Machine learning is composed of a group of algorithms including neural networks (NN), support vector machines (SVM), self-organizing maps (SOM), decision trees (Dtree), random forests (RF), case-based reasoning (CBR), k-nearest neighbours (k-nn) and genetic programming (GA) (Lary et al., 2015).

In this study, we develop a software framework for predicting drought and land fires risk areas based on spatial prediction of vegetation indices. The developed framework is a combination of machine learning algorithms that include ANN, SVM, RF, k-nn, CART and MARS with an algorithm compiled from the interpolation of Inverse Distance Weight. The use of machine learning in this framework aims to classify and predict drought indices from vegetation indices data that have spatial and temporal resolutions. The use spatial interpolation is an approach to determine numerical values in non-sampling areas. The software framework names are Satellite Imagery based Machine learning for the Index of Aridity (SIMIA). ‘Background’ in this paper discusses the proposed theoretical background in the framework, ‘Materials & Methods’ discusses the research methods, ‘Results’ discusses the results and discussions, ‘Conclusions’ discusses the conclusions, and the last section is the references.

## Background

ML (Machine Learning) is a subfield of artificial intelligence in which computers learn the relationship of a data set through a training process. The ML explores new relations of the location, date and other qualities data in a large data set based on the experiences from the historical data acquired (Huntingford et al., 2019; Fang et al, 2020). The ML can classify high dimensional data effectively and efficiently, map class of data which have very complex characteristics as in remote sensing (Zaraza Aguilera, 2020). In the field of remote sensing, the machine learning technique has been used to calculate identification and pattern recognition from a set of historical climate data and topography to produce information on land coverage changes. CART is an algorithm in machine learning that works based on the Decision Tree (DT). The core principle of this algorithm classifies a number of object data by divide them into both discrete binary data and continuous binary data as the predictable variables. The feature-selection of the classified objects used Gini Index (1), in which TS is the training data, $f \left( {K}_{i},TS \right) $ is the probability of data corresponds *to the K class* (Fang et al, 2020).

(1) \begin{eqnarray*}\mathrm{G}=\sum \sum _{\mathrm{j}\not = \mathrm{i}}^{} \left( \frac{\mathrm{f} \left( {\mathrm{K}}_{\mathrm{i}},\mathrm{TS} \right) }{ \left\vert \mathrm{TS} \right\vert } \right) \left( \frac{\mathrm{f} \left( {\mathrm{K}}_{\mathrm{j}},\text{TS} \right) }{ \left\vert \mathrm{TS} \right\vert } \right) \end{eqnarray*}

(2) \begin{eqnarray*}E=\sum _{i=1}^{c}-{p}_{i}\ast lo{g}_{2}({p}_{i})\end{eqnarray*}

*E* is an entropy calculated using the Gini Index using the Gini Index logarithmic. The concept of Entropy in the CART algorithm classifies a set of data using the characteristics that are in the data class (training data) (Kamal et al., 2000). The characteristic of this algorithm is when the Gini Index increases, the objects heterogeneity in the class is going to rise. Otherwise, when the Gini Index is decreasing, the object homogeneity in the class is increasing. CART is one of machine learning algorithms used for prediction using multidimensional Cartesian space with a function: $X=f \left( Y \right) +\epsilon $ where *Yϵ*ℜ^*d*^, *Xϵ*ℜ^*d*^. CART prediction is calculated by the function $\widehat{X}=\hat {f} \left( \hat {Y} \right) $. The notation *ϵ* represents various intrinsic data condition or diversity of data variables that are unpredictable or not observed. The disturbance in the prediction results is determined by a factor (*Y*) (Wang et al., 2004).

RF is a non-parametric ensemble classification and machine learning regression that uses DT as the basis for classification of data sets. It means that the mean prediction of multiple decision trees is trained using data obtained from different subsets but from the same data (Li et al., 2017). RF is an object classification method consisted of a group of tree-structured classifier, or it is denoted as $ \left\{ h \left( y,{\Theta }_{s} \right) k=1,2,\ldots \right\} $ in which $ \left\{ {\Theta }_{s} \right\} $ is the random vector distributed in each classifier node that is denoted as the y-class (Kullarni & Sinha, 2013). The RF equation is described as follow (3):

(3) \begin{eqnarray*}{T}_{s}={T}_{r} \left( s{|}{p}_{1},{p}_{2},\ldots ,{p}_{n} \right) = \frac{{\Pi }_{i}^{T}{T}_{r} \left( {p}_{1}{|}s \right) {T}_{r}(s)}{{T}_{r}({p}_{1},{p}_{2},\ldots ,{p}_{n})} \end{eqnarray*}

notation of ${T}_{r} \left( {p}_{i}{|}s \right) $ is the probability of input data on the s class. ANN is composed of a group of interconnected nodes and is called a neuron or neuronode. Interconnection between neuronodes can be described as the interconnection of a large number of simple processors arranged in layers (Raczko & Zagajewski, 2017; Akhand, Nizamuddin & Roytman, 2018). The equation used is (4):

(4) \begin{eqnarray*}{\gamma }^{k} \left( {t}_{s} \right) ={f}_{0} \left[ \sum _{j=1}^{m}{w}_{kj}.{f}_{n} \left( \sum _{i=1}^{n}{w}_{ji}{x}_{i} \left( {t}_{s} \right) + \left( w{j}_{0} \right) \right) +w{k}_{0} \right] \end{eqnarray*}

Where *n* is the number of data input variables, *m* is the number of neurons, *x*_i_(*t*) is the *i*^th^ of input variable at time *ts*, *w*_ji_ is the weight of the *i*^th^ of variable neuron which is interconnected with the *j*^th^ neuron, *wj*_0_ is biased for neuron at *j*, *fn* is a function of neuron activation, *wkj* is the variable weight of neuron at *j* and neuron at *k*, *wk*_0_ is biased for external neuron *k*, *f*_0_ is an activation function for external neuron, and ${\gamma }^{k} \left( {t}_{s} \right) $ is the external neuron *k* which is predicted at time *t*_*s*_ (Belayneh & Adamowski, 2013).

SVM is a method that has been implemented to classify tree types of hyperspectral data. SVM works by using training data to find the most optimal hyperplane of attribute classes. It can be simply described that the optimal hyperplane is determined from the margin value between the two training data classes. If a hyperplane cannot be determined, a kernel function is used to convert the original data into a higher dimension.

The k-nn algorithm is a non-parametric method that is widely used for classification in pattern recognition. The main principle of k-nn is that the category of data point is determined according to the classification of neighbour *k* closest to that point. As an illustration, an arbitrary point which is a subset in the training set *S = {(p*_1_*, q*_1_*), (p*_2_*, q*_2_*), …(p*_*N*_*, q*_*N*_*)}* with the number of attributes or entities *N*. The value of *p*_*i*_ *ϵR*^*d*^ is a vector feature and *q*_*i*_*ϵy* = *a*_1_, *a*_2_…*a*_*M*_ as the classification label, *i* = 1, 2, …, *N*. If there is input data *p* then the value of k-nn or *N*_*k*_(*p*) is the distance of the training set entities (Guo et al., 2018). To calculate k-nn, Euclidean Distance should be determined first using (5):

(5) \begin{eqnarray*}d \left( p,q \right) =\sqrt{\sum _{i=1}^{n}({p}_{1}-{q}_{1})^{2}}\end{eqnarray*}

The notation *d* is used for the Euclidean distance, the notation *p* is used for the training data set, and the notation for the testing data set is *q*. The distance between the training data was compared in order to determine the closest distance.

MARS is a non-parametric multivariate regression analysis that uses a linear regression approach one by one for each variable, whereas if the variable is non-linear then it is approximated by using the slope interval of the variable. MARS analyses sequentially and looks for interactions between variables (Yang et al., 2016). The MARS equation is (6):

(6) \begin{eqnarray*}f \left( x \right) =\sum _{i=1}^{k}{c}_{i}{b}_{i}(x)\end{eqnarray*}

Which *b*_*i*_ is the basis of the linear function, and *c*_*i*_ is the constant coefficient.

VI is a quantitative value of the measurement of vegetation canopy in receiving and reflecting the spectrum of light and interpreted as a spectral characteristic of vegetation (Paseleng, Joko Prasetyo & Hartomo, 2018). Formulation VI that is used for drought monitoring is classified into four groups name (Du et al., 2018; Wang et al., 2018): (1) Greenness Related Vegetation Indices that includes NDVI and VCI ; (2) Temperature Related Vegetation Indices such as the TCI ; (3) Combined Vegetation Indices, such as the VHI ; (4) Burn Severity Indices, such as NBR, and BAI, CSI. The VI in the proposed framework are as presented in Table 1.

**Table 1:** The VI in the proposed framework.

VI	Equation	Description	Ref.
*NDVI*	$ \frac{{\rho }_{NIR}-{\rho }_{red}}{{\rho }_{NIR}+{\rho }_{red}} $	NDVI is a vegetation index that uses a combination of NIR and RED spectrum reflectance to measure vegetation greenness. Chlorophyll and carotenoid elements of a plant absorb more red spectrum with *λ* of 0.66 µm, and *ρ*_*NIR*_ with *λ* of 0.86 µm. NDVI represents photosynthesis, biomass, dominant species and biogeochemical processes.	Hashim, Abd Latif & Adnan (2019)
*VCI*	$ \frac{NDV{I}_{a}-NDV{I}_{min}}{NDV{I}_{max}+NDV{I}_{min}} \times 100$	VCI is used as an indicator to identify changes in vegetation from bad condition to good condition. NDVImin and NDVImax are the mean values of seasonal NDVI. NDVI represents the dynamics of seasonal vegetation, while VCI reflects relative changes in humidity conditions from very bad to optimal condition.	Garnero & Godone (2013)
*TCI*	$ \frac{LS{T}_{max}-LS{T}_{a}}{LS{T}_{max}+LS{T}_{min}} \times 100$	TCI is an indicator of drought that gives an indication of stress on vegetation caused by high temperature and excessive humidity. *LST*_*max*_ or Land Surface Temperature maximum is the highest average temperature of the current month and *LST*_*min*_ is the lowest average temperature of the current month.	Angka (2019)
*VHI*	$\alpha VCI+ \left( 1-\alpha \right) TCI$	VHI is a vegetation index which is a combination of VCI and TCI. VCI represents humidity in the vegetation canopy which absorbs visible light and NIR spectra. TCI represents the air temperature in the vegetation canopy which absorbs the IR spectrum. VHI is a vegetation index that represents the level of air temperature (TCI) and soil moisture (VCI). A lower VHI value indicates a high drought while a higher VHI value indicates a wet or no drought condition.	Shi et al. (2020)
*NBR*	$ \frac{{\rho }_{NIR}-{\rho }_{SWIR}}{{\rho }_{NIR}+{\rho }_{SWIR}} $	Normalized Burn Ratio (NBR) is the ratio between NIR and SWIR with both *λ* at 2.08–2.35 µm. This index is used to identify a burnt area after a fire incident and provide a quantitative measure of the severity of the burned area.	Verdin et al. (1999)
*BAI*	$ \frac{1}{{ \left( {\rho }_{cr}-{\rho }_{r} \right) }^{2}{ \left( {\rho }_{cnir}-{\rho }_{nir} \right) }^{2}} $	BAI is a special index to distinguish areas affected by fire. This index is calculated from the spectral distance from each pixel to the reference spectral point, where the newly burned area tends to coalesce.	Tripathi et al. (2013)

The class of vegetation status of NDVI in LANDSAT 8 OLI are as presented in Table 2A (Hashim, Abd Latif & Adnan, 2019).

**Table 2:** The class vegetation status of NDVI (A) and class of indicator VHI, TCI, VCI (B) (Hashim, Abd Latif & Adnan, 2019).

**(A)**		
Vegetation Class	Description	NDVI Value
Non Vegetation	Barren areas, build up area, road network	−1 to 0.199
Low Vegetation	Shrub and grassland	0.2 to 0.5
High Vegetation	Temperate and Tropical urban forest	0.501 to 1.0

**(B)**	
Vegetation Status	VHI
Extreme Drought	<10
Severe Drought	10–20
Moderate Drought	20–30
Mild Drought	30–40
No Drought	>40

The IDW method is used to determine the non-observation point value, which is calculated based on the weight and distance of the closest observation point. Determination of IDW is done using (7) (Garnero & Godone, 2013).

(7) \begin{eqnarray*}Z= \frac{\sum _{i=1}^{n} \frac{{z}_{i}}{{ \left( {h}_{ij}+\delta \right) }^{\beta }} }{\sum _{i=1}^{n} \frac{1}{{ \left( {h}_{ij}+\delta \right) }^{\beta }} } \end{eqnarray*}

The notation *Z* is the value of observation point that will be calculated or sought, the notation *Z*_*i*_ is the value of observation point that is known based on the calculation, and the notation *β* is the weight factor and *δ* is the smoothing factor. The value of *h*_*ij*_ is the distance between the known and unknown sample points and is determined using the Euclidean equation as shown below (8).

(8) \begin{eqnarray*}{\mathbi{h}}_{\mathbi{ij}}=\sqrt{(\Delta \mathbi{x})^{2}-(\Delta \mathbi{y})^{2}}\end{eqnarray*}

where Δ*x* and Δ*y* are the distance between unknown point at *j* and sample one at *i*.

## Materials & Methods

This study uses monthly LANDSAT 8 OLI (Operational Land Imager) imagery data from 2018 to 2019. The data source is the United States Geological Survey (USGS) https://earthexplorer.usgs.gov/ on path/row 120/65. LANDSAT 8 OLI imagery data consists of 11 bands with different bandwidth and resolution (Table 3).

**Table 3:** Band name, bandwidth and resolution of LANDSAT 8 OLI.

Band	Bandwidth	Resolution
Band 1 Coastal	0.43–0.45	30
Band 2 Blue	0.45–0.51	30
Band 3 Green	0.53–0.59	30
Band 4 Red	0.64–0.67	30
Band 5 NIR	0.85–0.88	30
Band 6 SWIR 1	1.57–1. 65	30
Band 7 SWIR 2	2.11–2.29	30
Band 8 Pan	0.50–0.68	15
Band 9 Cirus	1.36–1.68	30
Band 10 TIRS 1	10.6–10. 19	100
Band 11 TIRS 2	11.5–12.51	100

**Figure 1:**
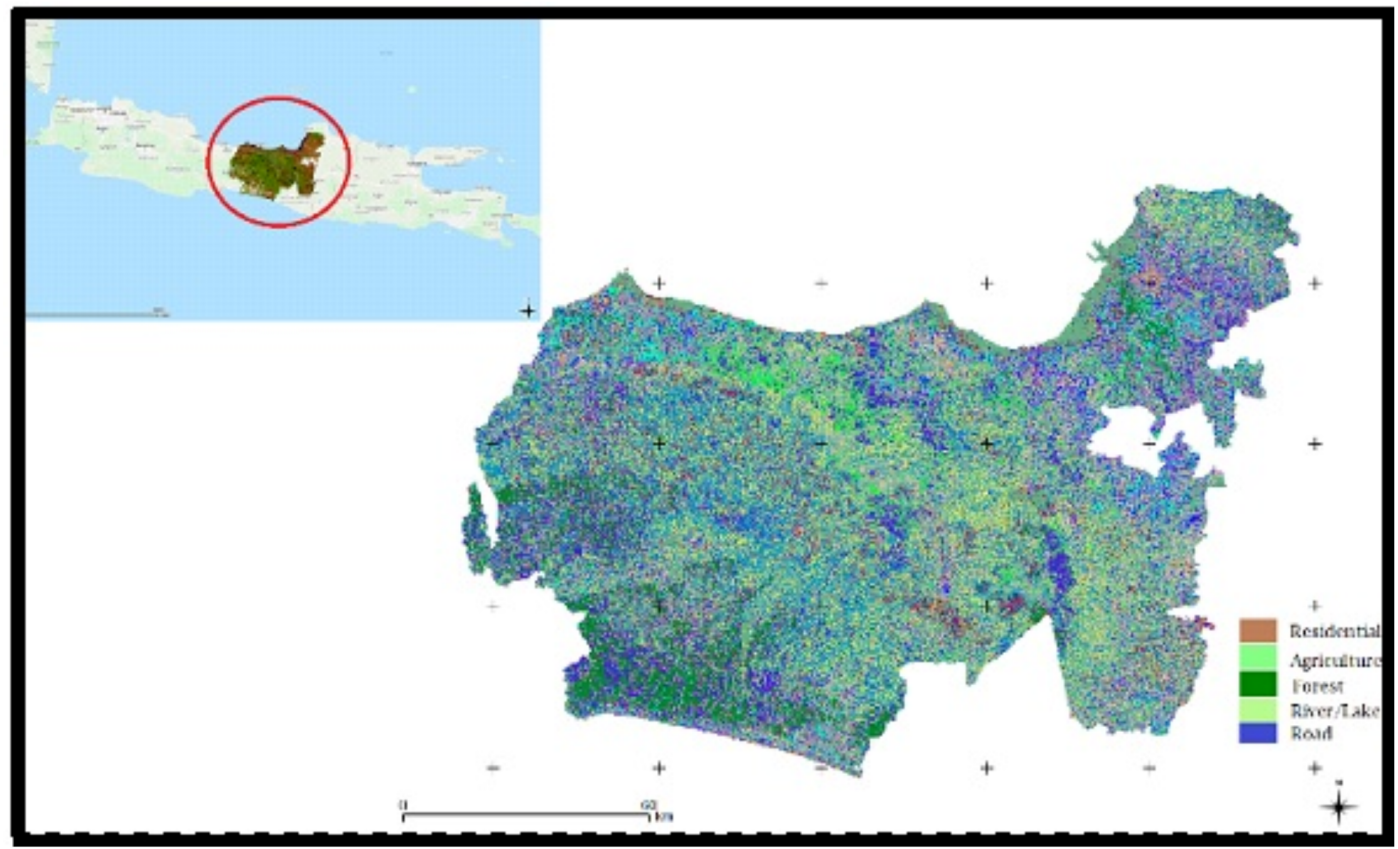
The land cover study area of Central Java Province of Indonesia.

This study was conducted in Central Java Province, Indonesia covering 346 sub-districts. Geographically the study area is in the coordinates of 5°40′ and 8°30′South Latitude and 108°30′  and 111°30′. The air temperature at the study area was between 23.5 °C to 27.8 °C, the humidity was between 66% and 83% and the highest rainfall was 3,143 mm (Badan Pusat Statistik, 2019). The proposed framework runs in stages as shown in Fig. 1 below. Land cover in the study area varies but is dominated by residential areas, public and industrial facilities, Agriculture includes rice fields, fields and plantations, Forest, River and Road.

**Figure 2:**
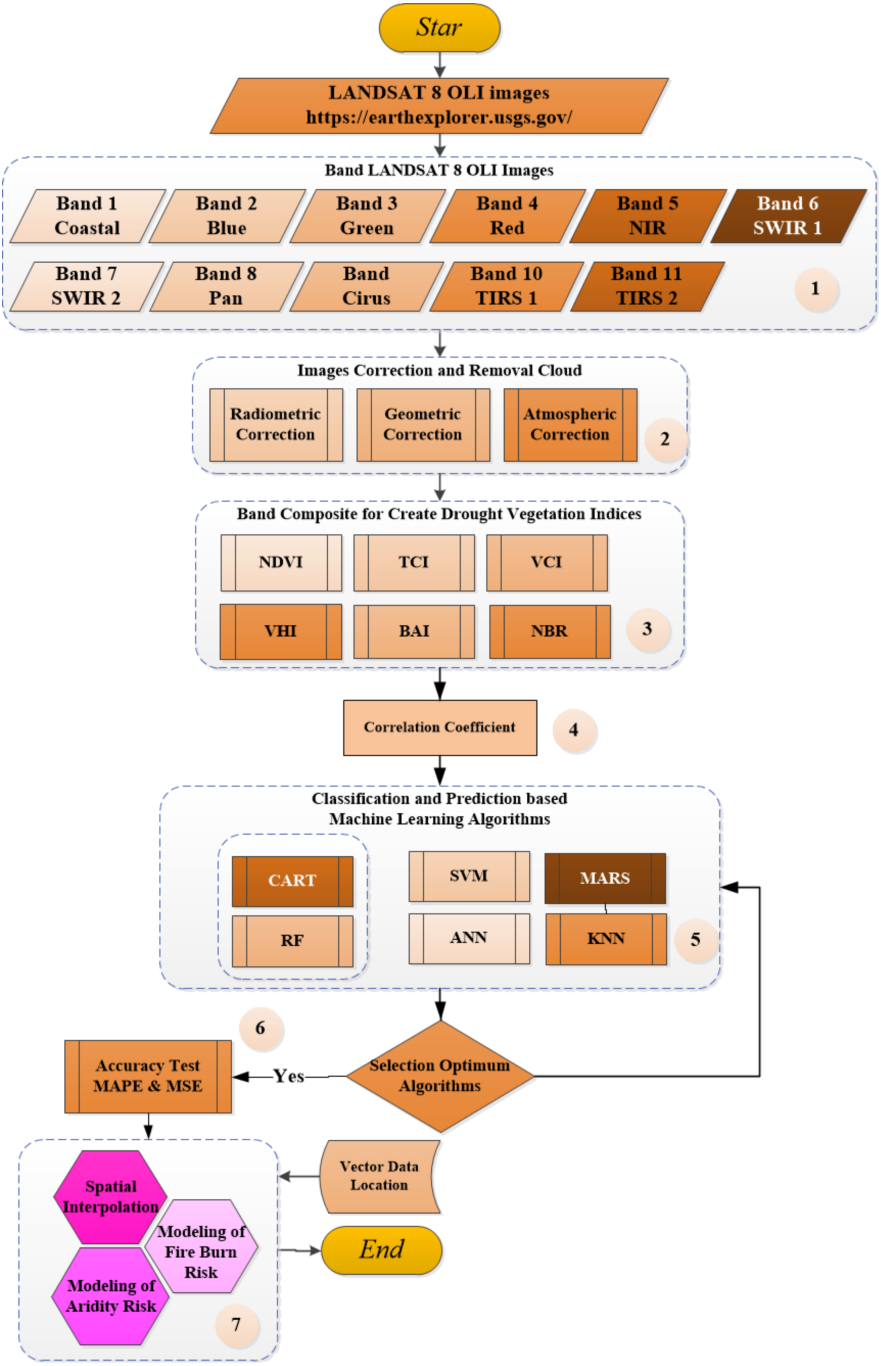
The proposed framework in the study.

The proposed framework runs in stages as shown in Fig. 2 below. (1) The LANDSAT 8 OLI image is composed of 11 bands in which each band has different wavelength and function (Table 3). The LANDSAT 8 OLI images obtained from https://earthexplorer.usgs.gov/. (2) The process of geometric correction, radiometer correction and atmospheric correction. (3) Combining bands and calculating use mathematical formulations as in (4)–(10) to form vegetation indices. The generated vegetation indices data with numeric data types. (4) Correlation analysis and temporal regression of vegetation index data were performed to see the correlation and trend of vegetation indices at the same location from time to time (monthly). Each vegetation data indices are divided into two categories i.e., testing data at 70% and learning data at 30% of the total data. (5) Classifying and predicting vegetation indices data using ANN, SVM, RF, k-nn, CART and MARS algorithms. Choose the most optimal algorithm for analysing vegetation index data. (6) Modelling of drought risk areas, forest fire risk areas and spatial connectivity between areas with high risk of drought and land fires using the Getis Ord method. Spatial prediction is done to determine the risk value of drought and land fires in non-sampled areas. (7) Testing of the interpretation of machine learning prediction results imagery with real conditions in the field using the Confusion Matrix method.

The eminences of this framework are: implementing ML to predict a period of the vegetation index value as a data base to determine the drought risk area, and generating information about the drought risk area in the smallest area using the IDW spatial interpolation method. This interpolation method provides a function to spatially predict areas which are not the observation points.

## Results

The correlation analysis of VI data aims to see the distribution, correlation and direction of the VI data trend in a certain temporal value. Correlation analysis of the observed VI was done using BAI, NBR, NDVI, TCI, VCI and VHI in 2018 and 2019 which was visualized in the form of scatterplot matrices.

**Figure 3:**
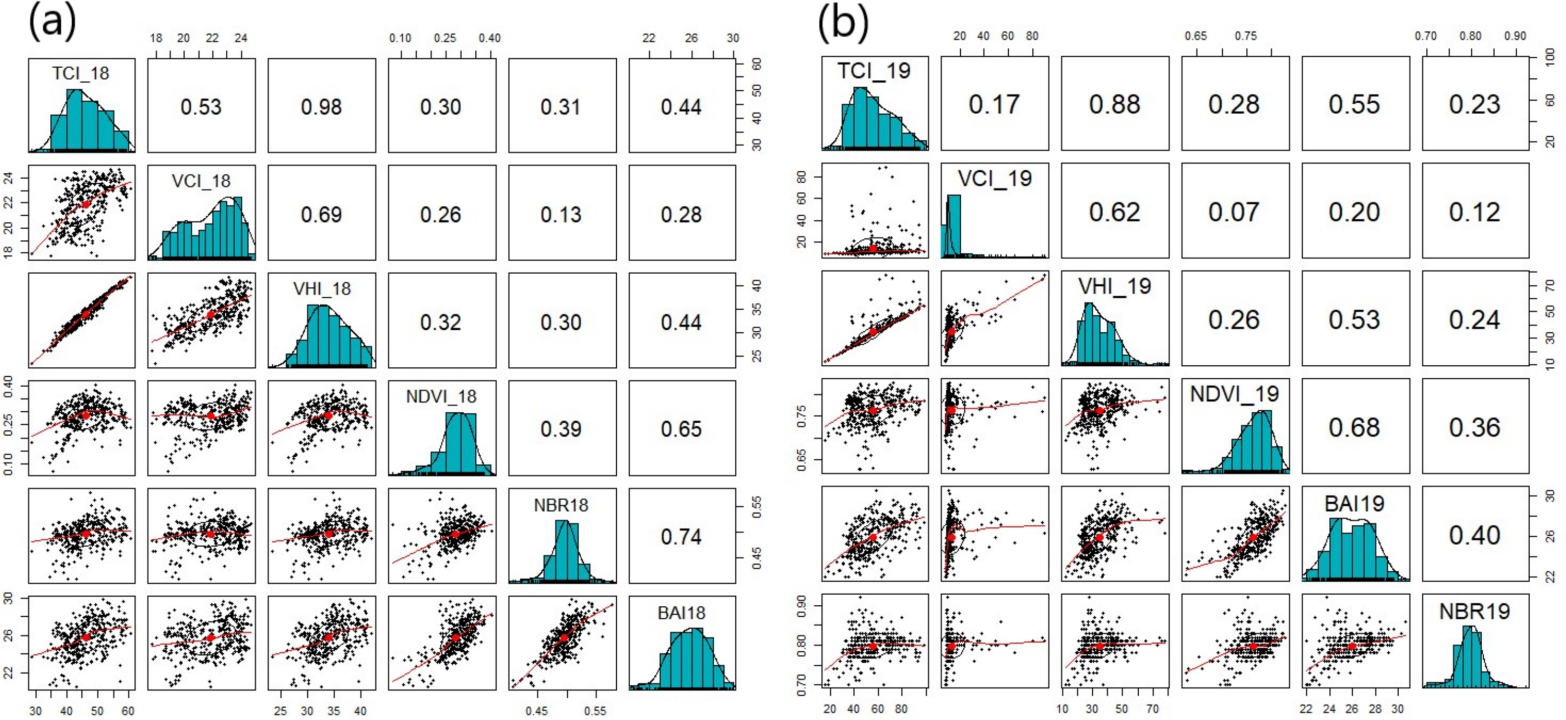
Analysis of the Vegetation Indices Association in 2018 (A) and 2019 (B).

The analysis using Pearson Correlation Coefficient method shows that the association between vegetation indices is very strong as indicated by the magnitude of the coefficient *r*, which is <0.5 or <−0.5, and which some with values >0.3 or >−0.3 are considered as intermediate association and some with values <0.3 or <−0.3 are considered as weak association (Figs. 3A and 3B).

On all VI (Figs. 3A and 3B), line curves represent data distributions (black dots), data centers (red dots) and data trends (red lines) that describe correlation directions i.e., linear and linear curve. Bar chart for each index shows that data is normally distributed in each sample space. VI that correlates with the Greenness Related Vegetation Indices class shows a linear correlation–an increase in the value of an index will be followed by other indices, while VI that correlates with Burn Severity Indices shows a linear curve correlation, which means an increase in the value of an index will be followed by other indices. However, up to certain point, it will tend to decrease. Meanwhile, VHI is formed by VCI and TCI data, so that it has a moderate to strong correlation. The trend of VHI and VCI and TCI shows a linear pattern. Linear curve pattern is observed between VI in the Burn Severity Indices class and other vi. The difference in correlation analysis results on the analyzed VI indicates that each VI has a different sensitivity towards noise (background canopy and atmospheric effect) at the same location.

The VI, which is the drought indicator, is a combination of NDVI (vegetation stress), VCI (time, duration, and drought intensity), TCI (vegetation stress triggered by temperature), and VHI (vegetation stress caused by wetness) (Jayawardhana & Chathurange, 2020). The VI is used as a drought-risk indicator as it has strong correlation with precipitation, vegetation canopy greenness, canopy and soil water content, also land covers (Shi et al., 2020). The *VI* prediction is done by using machine learning, especially on RF and CART algorithms and comparison is made with ANN, k-nn, SVM, and MARS. Results of *VI* prediction are useful for identifying the risk of drought and land fires in an area. The *VI* prediction is done by using machine learning, especially on RF and CART algorithms and comparison is made with ANN, k-nn, SVM, and MARS.

The results of the RF accuracy analysis and test are shown in Table 4A. The validation testing of prediction results using MAPE shows a low average difference between the predicted results from survey data at 1.04% in all indices. The test using MSE aims to see the level of similarity between the prediction results with survey data, which the smaller and near zero shows the higher similarity. The average RMSE in this study is 0.05.

**Table 4:** Prediction of vegetation index using the RF algorithm and the CART algorithm in 2019–2020.

VI_*Pred*_			VI_*Survey*_		MAPE	MSE
VI	Min	Max	Min	Max		
**(A)**						
NDVI	0.389	0.597	0.049	0.405	0.500	0.071
TCI	22.359	78.912	22.359	78.912	0.000	0.000
VCI	13.678	43.638	10.023	57.037	23.492	23.736
VHI	21.375	52.375	17.740	59.987	14.121	3.954
BAI	21.261	30.193	21.215	30.210	0.056	0.000
NBR	0.564	0.716	0.552	0.749	4.406	0.000
**(B)**						
NDVI	0.426	0.583	0.246	0.583	0.000	0.000
TCI	30.387	71.577	84.639	71.557	0.028	0.000
VCI	14.010	37.593	14.010	38.000	0.003	0.166
VHI	22.503	51.655	22.503	52.000	0.019	0.119
BAI	21.938	28.755	21.938	29.000	0.003	0.060
NBR	0.557	0.706	0.577	0.658	7.295	0.002

The prediction results of vegetation index using the CART algorithm shown in Table 4B. Testing the validation of prediction results using MAPE shows the average difference between the predicted results with survey data is very low at 1.05% in all indices. The average RMSE in this study is 0.05. The result of this experiment indicates that CART algorithm is able to accurately predict vegetation index data.

Validation testing of prediction results using MAPE shows that the average difference between the prediction results and survey data is very low at 5.78% for all indices. The average RMSE in this study is 2.19. The results of this experiment indicate that the ANN algorithm can accurately predict vegetation index data.

The results of vegetation index prediction using k-nn are shown in Table 5B. The validation testing of prediction results using MAPE shows that the average difference between the prediction results and survey data is very low at 6.07% in all indices. The average RMSE in this study is 2.37. The results of this experiment indicate that the k-nn algorithm can accurately predict vegetation index data. The SVM algorithm works by classifying input data to form a multidimensional hyperplane. Hyperplane multidimensional distinguishes vegetation index variable classes namely NDVI, TCI, VCI, VHI, BAI and NBR.

**Table 5:** (A) Prediction of vegetation index 2019–2020 using the ANN algorithm and the (B) knn algorithm.

VI_*Pred*_			VI_*Survey*_		MAPE	MSE
VI	Min	Max	Min	Max		
**(A)**						
NDVI	0.331	0.602	0.348	0.615	2.114	0.000
TCI	27.465	76.772	22.359	79.531	3.469	7.612
VCI	13.210	55.443	13.032	57.037	2.795	2.541
VHI	19.801	40.861	17.740	41.711	11.893	0.723
BAI	20.979	30.492	21.214	30.210	0.933	0.080
NBR	0.555	0.695	0.552	0.749	7.210	0.003
**(B)**						
NDVI	0.396	0.613	0.348	0.615	0.325	0.002
TCI	26.217	77.847	22.359	79.351	1.895	14.884
VCI	13.098	41.098	13.023	57.036	27.944	0.006
VHI	18.591	53.441	17.740	59.591	10.320	0.724
BAI	21.289	30.012	21.220	30.012	0.000	0.005
NBR	0.555	0.734	0.552	0.749	2.003	0.002

The SVM kernel uses the difference between values to differentiate classes in multidimensional data. For example, if there is a TCI data class, two dimensional spaces will be formed, separated by a hyperplane as a straight line. In vegetation index classes, linear hyperplanes are not easy to form because there are some values that are difficult to include in these classes. The solution is to add a dimension using polynomial, sigmoid, and radial basis kernel functions (Table 6A).

**Table 6:** Prediction of vegetation index using SVM Algorithm (A) and MARS Algorithm (B) in 2019–2020.

VI_*Pred*_			VI_*Survey*_		MAPE	MSE
VI	Min	Max	Min	Max		
**(A)**						
NDVI	0.351	0.617	0.040	0.504	22.421	0.045
TCI	23.569	78.495	22.358	79.351	1.079	0.732
VCI	13.276	56.479	13.023	57.036	0.977	0.310
VHI	18.701	59.289	17.740	55.711	16.915	12.802
BAI	21.390	29.835	21.213	30.210	1.241	0.140
NBR	0.552	0.741	0.552	0.749	1.068	0.003
**(B)**						
NDVI	0.359	0.615	0.049	0.405	21.782	0.044
TCI	30.387	71.557	22.359	75.912	9.321	18.966
VCI	13.023	57.037	13.023	57.037	0.000	0.000
VHI	17.740	59.987	17.740	59.987	0.000	0.000
BAI	21.261	30.193	21.215	30.210	0.056	0.000
NBR	0.551	0.726	0.552	0.749	3.071	0.001

Validation testing of prediction results using MAPE shows that the average difference between the prediction results and survey data is very low at 6.28% in all indices. Tests using MSE aims to see the level of similarity between the results of predictions and survey data, the smaller and near zero, the higher similarity. The average RMSE in this study is 2.01. The results of this experiment indicate that the SVM algorithm can accurately predict vegetation index data.

The prediction results using MARS algorithm can be seen in Table 6B. Validation testing of prediction results using MAPE shows that the average difference between the predicted results and survey data is very low at 5.34% in all indices. Tests using MSE aims to see the level of similarity between the results of predictions and survey data, the smaller and near zero, the higher similarity. The average RMSE in this study is 4.15. The results of this experiment indicate that the MARS algorithm can accurately predict vegetation index data.

The experimental results show that the most efficient algorithms in predicting vegetation index are RF and CART. RF has a difference between the predicted results and 1.04% survey data (MAPE) and the smallest value close to zero is 0.05 (MSE). CART has a difference between the predicted results and 1.05% survey data (MAPE) and the smallest value close to zero is 0.05 (MSE) (Table 7).

**Table 7:** Average MAPE and MSE on the Machine Learning Algorithm Test.

Algorithms	MAPE (%)	MSE
ANN	5.70	2.19
k-nn	6.07	2.07
SVM	6.28	2.01
RF	1.04	0.05
CART	1.05	0.05
MARS	5.34	4.15

**Figure 4:**
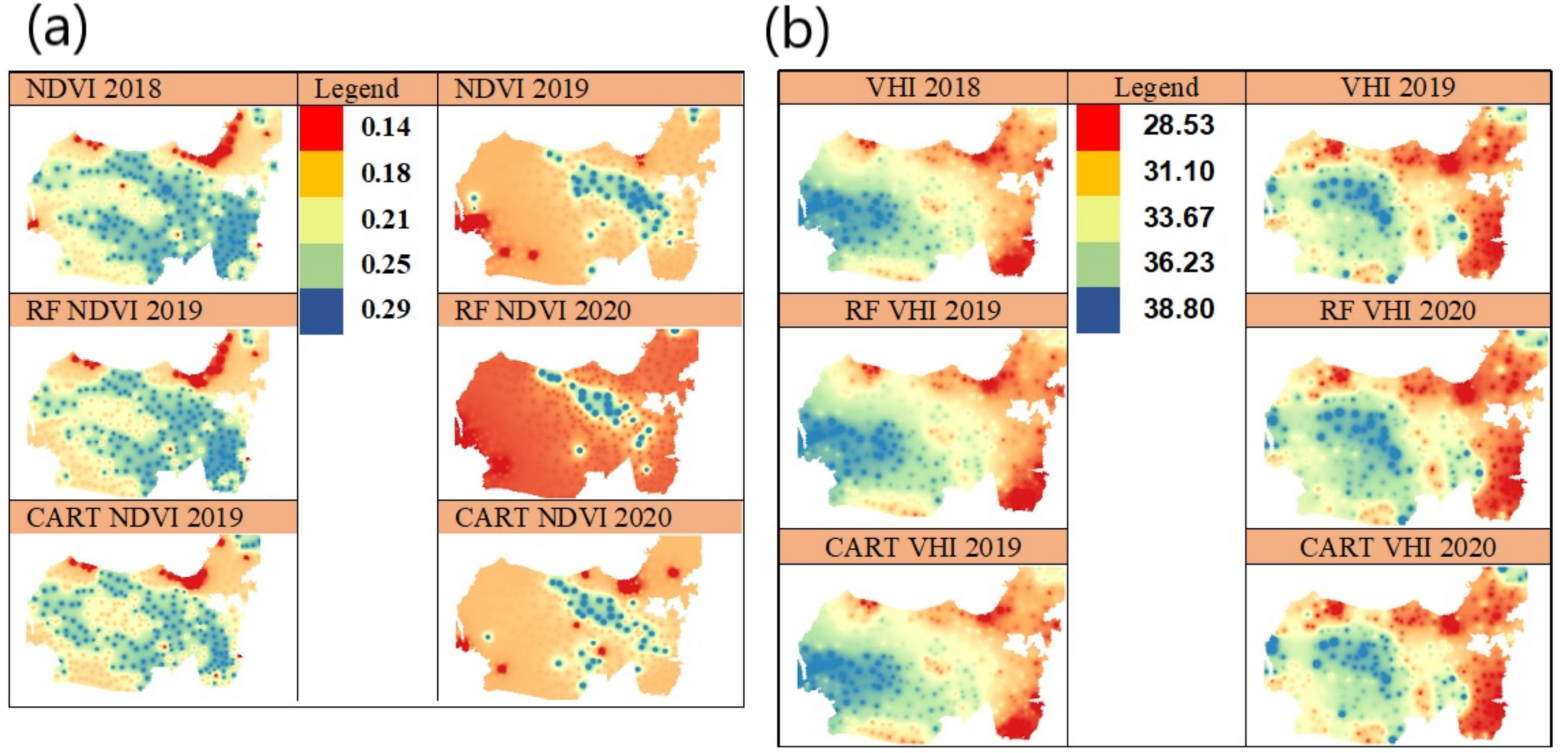
Spatial prediction using IDW in 2019–2020 of NDVI vegetation index (A) and spatial prediction of VHI vegetation index (B).

Based on the results of the machine learning algorithm validation test, it can be seen that the most effective and efficient methods are RF and CART (Table 7). The next discussion presents an interpolation of the value of vegetation index and its status at a non-sample point of special survey on RF and CART. Figure 4 shows the spatial prediction of NDVI in 2019 to 2020 with IDW method. The experimental results show that in 2019, most regions in Central Java have high vegetation land coverage with NDVI >0.21. High NDVI is an indicator of high chlorophyll capacity contained in mesophyll canopy leaves. In contrast, in 2020, it is revealed that most regions in Central Java have low vegetation land coverage with NDVI <0.21. The low NDVI value is an indicator of vegetative pressure which has an impact on reducing chlorophyll content. Based on the spatially prediction, the NDVI dynamics between 2018 and 2019, can be inferred that the green vegetation biomass, LAE, photosynthesis activities, chlorophylls density in the vegetation canopy, and the amount of annual and monthly rainfall in 2020 are very low (Verdin et al., 1999). On Fig. 4A, it can be seen that the area which has low level of greenness is very wide, with NDVI <0.21, which means that there is stress pressure in the vegetation canopy as the result of low precipitation level, reduced water content in the canopy, and low water level in the soil. The prediction results show that the drought risk consistently happened in the most of Central Java area, and, in the contrary, the areas that did not experience drought are also consistent since 2018.

**Figure 5:**
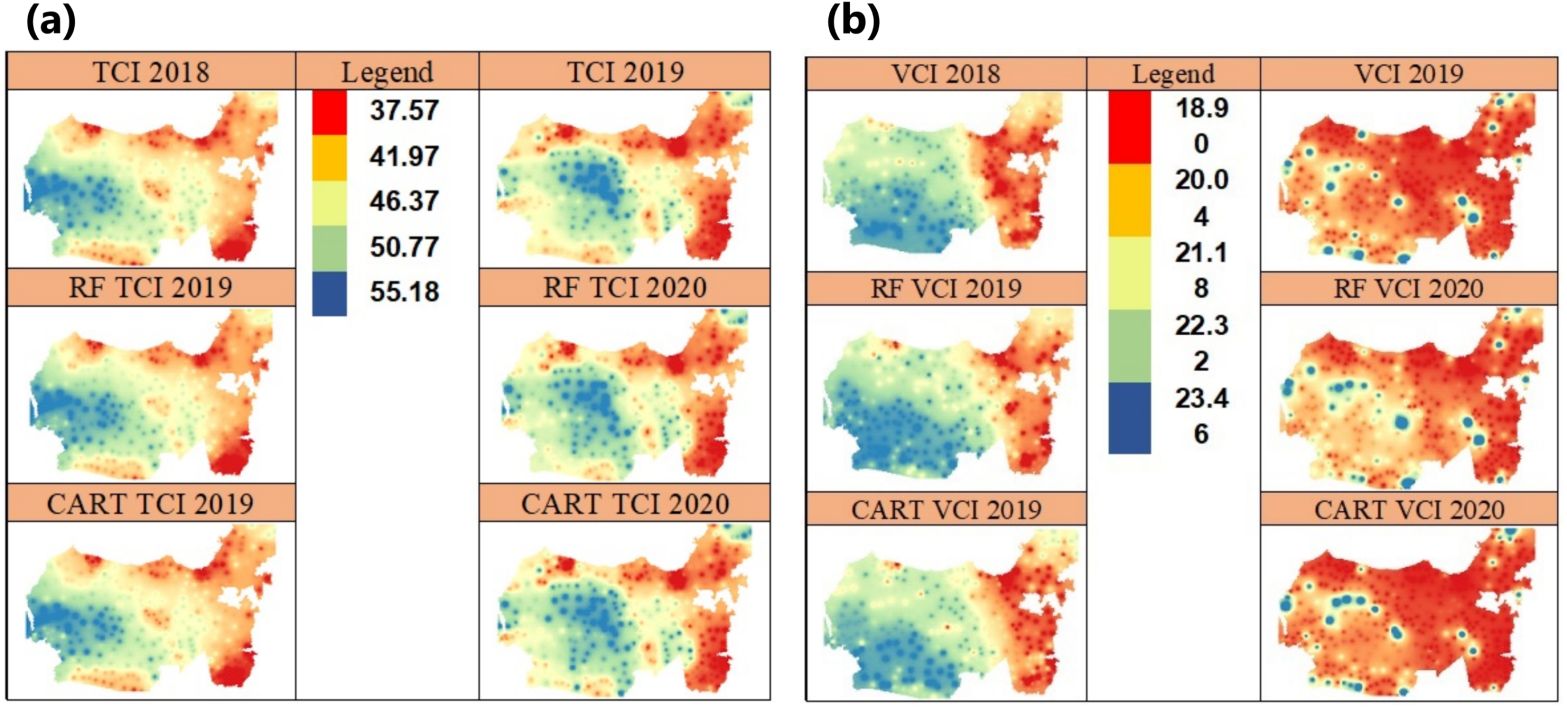
Spatial prediction in 2019–2020 using the IDW of TCI vegetation index (A) and the VCI vegetation index (B)

VHI consists of VCI, TCI, and *α* components which are the parameters to quantify VCI and TCI contributions. VCI is composed from NDVI value, while the TCI is composed of LST value, which all of them are calculated using monthly data time series period between 2018 and 2019.Vegetation land coverage can be seen from the dominance of regions that do not indicate drought with a VHI value >33.67 in 2019. In 2020 shows a decrease in the areas that have land coverage with thick vegetation and an increase in the areas which indicate drought shown by a VHI value <31.10 (Figs. 4B). Figure 4B shows that the VHI prediction from 2019-2020 is very low, which means that the vegetation in the most of study areas contain chlorophyll, the low leaf equivalent water thickness and LEA, mainly caused by low precipitation, and this condition indicates drought biophysically (Tripathi et al., 2013). VHI is composed of two components, namely, TCI index which is a thermal indicator and VCI index which is an indicator of soil surface moisture. TCI value describes the thermal surface of a ground caused by solar radiation reflectance. The TCI value is calculated based on the land surface temperature (LST) analyzed from the time series imagery pixel (Karamzadeh et al., 2017). The low TCI value is an indicator of an increased stress and vegetative growth pressure associated with drought phenomena (Gidey et al., 2018). Figure 5A shows that TCI can be accurately predicted using RF and CART algorithms and visualized spatially with IDW.

VCI is an indicator of drought level based on soil surface humidity. The VCI >50% represents normal conditions; between 35%–50% represents moderate drought and <35% represents high drought (Fig. 5B (Dutta et al., 2015)).

VCI that is measured in seasonal or annual terms in time series showing a value <20% indicates the occurrence of long drought. RF and CART algorithms can be used to accurately predict TCI using IDW function as shown in Fig. 5B. Figure 5B shows the potential for high to extreme drought i.e., <20.04. In this study, three indicators are used to see the areas before and after land fires as a result of long drought using BAI and NBR. Fire detection can be done through pixel extraction by identifying R, NIR and SWIR signals. Signal R can show contrasting color patterns and separate pixels that represent healthy vegetation and non-vegetation lands based on chlorophyll concentrations in plants. The newly burnt area reflects very low NIR and R signals and reflects a very high SWIR signal.

**Figure 6:**
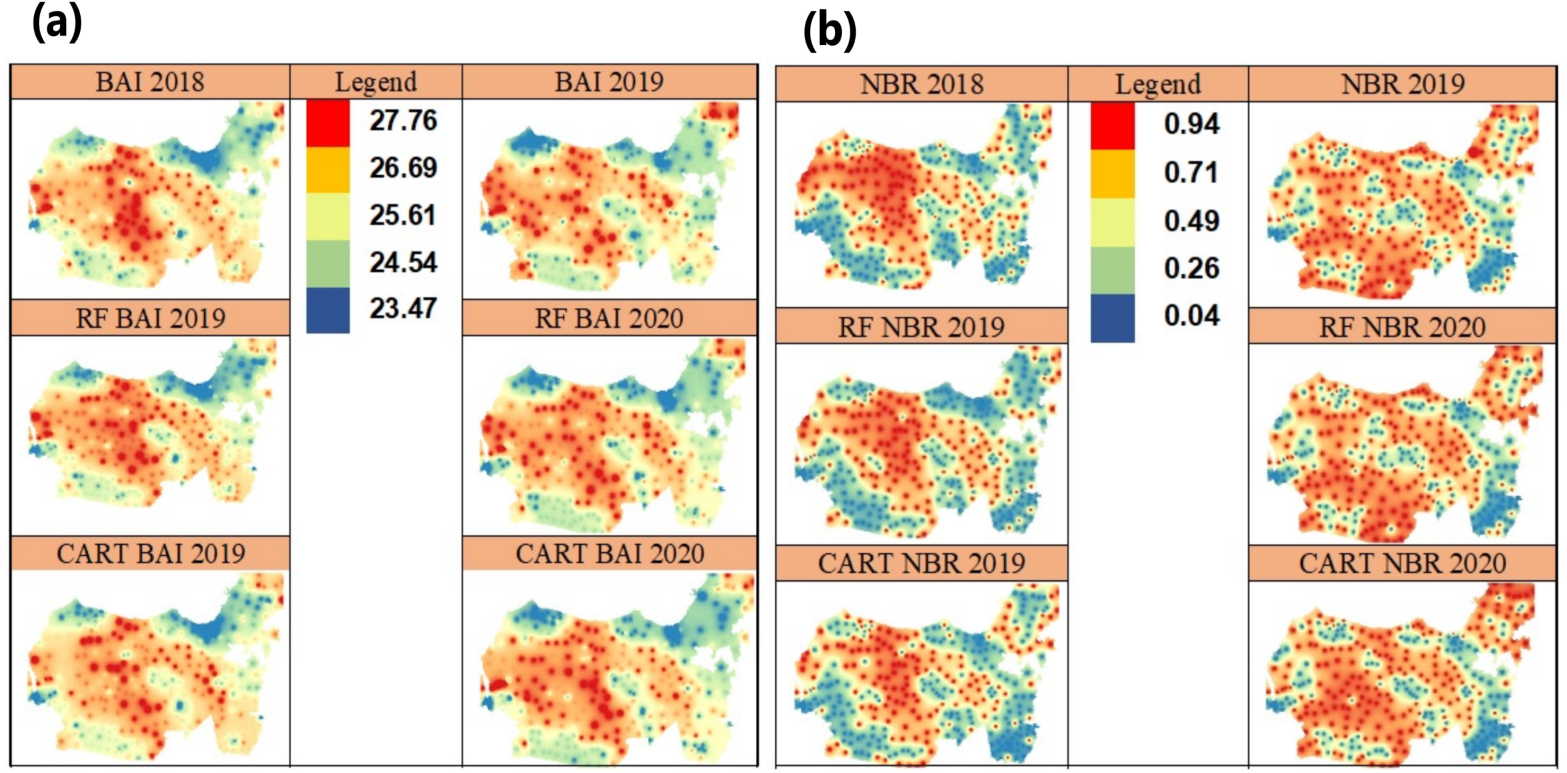
Spatial prediction of the BAI vegetation index (A) and the NBR vegetation index in 2019–2020 using IDW (B).

BAI is a vegetation index that has a threshold value which is calculated based on pixel comparison on time series that represents burnt and unburned areas (Fig. 6A) (Dalposso et al., 2013; Chuvieco et al., 2013). BAI is the latest method which has a high sensitivity towards the influence of soil types and the existing land use on the surface of the earth (Table 8A).

The computational experiment shows that the study area has a ΔBAI value of between −3.971 to −2.376, which is interpreted as a region where there are no land fires. This region shows the growth of new vegetation. Prediction using machine learning shows the algorithm that produces the closest value to ΔNBR1819 survey data which is ΔSVM and ΔCART algorithms (Table 8B). NBR is a vegetation index that is used to detect fires based on spectral changes which are interpreted as changes in the amount of chlorophyll content in plants, moisture, and charcoal or ash in the soil (Parks, Dillon & Miller, 2014).

**Table 8:** (A) Prediction of BAI values with Machine Learning algorithm and (B) ΔNBR values with Machine Learning algorithm.

**(A)**			
Algorithms	Min	Average	Max
ΔANN	−11.787	0.074	3.845
Δk-nn	−6.037	−0.016	6.742
ΔSVM	−3.617	−0.242	2.506
ΔRF	−2.647	−0.272	5.208
ΔCART	−3.067	−0.283	2.376
ΔMARS	−6.923	−0.283	5.028
ΔBAI1819	−3.971	−0.216	2.376
**(B)**			
ΔANN	−0.426	−0.362	−0.204
Δk-nn	−0.402	−0.302	−0.212
ΔSVM	−0.410	−0.301	−0.210
ΔRF	−0.381	-0302	−0.237
ΔCART	−0.410	−0.303	−0.244
ΔMARS	−0.397	−0.302	−0.236
ΔNBR1819	−0.412	−0.303	−0.208

The ΔNBR is VI which shows energy differences between healthy vegetation areas and stressed vegetation areas caused by water stress as a result of fire and is used to determine the potential of regrowth. The severity degree of the burned areas is calculated based on the differences between before the fire and post-fire vegetation in the same area. ΔNBR aims to normalize spatial variation in vegetation cover before the fire as this method is very accurate in other studies (Wulder et al., 2009). The severity degree is notated with *differenced (d)* or Δ or *delta*. The result of Δ calculation shows that the severity level, vegetation survival, and delayed vegetation mortality. The NBR value is between +1 and −1, and the higher the value shows the burned areas. The experimental results show that the study area has a ΔNBR value of between −0.020 to −0.412 which is interpreted as a region that has just begun to grow. Prediction using machine learning shows two ΔSVM and ΔCART algorithms that produce the closest value to ΔNBR1819 survey data (Fig. 6B), (Table 8B). The ΔNBR which is calculated from 2018 to 2019 shows a typical pattern of no fire and pattern of lands that start to be vegetating which can reflect signal and is depicted on image pixel.

## Conclusions

The correlation coefficient analysis concerning the results of vegetation index observation namely, BAI, NBR, NDVI, TCI, VCI and VHI in 2018 and 2019 shows that the association between vegetation indices is very strong, with coefficient *r* is <0.5 or <−0.5, some of which have values >0.3 or >−0.3 as medium association and some have values <0.3 or <−0.3 as weak association. The experimental results show that the most efficient algorithms in predicting vegetation index are RF and CART. RF has a difference between the predicted results and 1.04% survey data (MAPE) and the smallest value close to zero is 0.05 (MSE). CART has a difference between the predicted results and 1.05% survey data (MAPE) and the smallest value close to zero is 0.05 (MSE). Spatial prediction shows that the vegetation index of machine learning prediction result data is almost the same size as the data from field observations. NDVI indicates high vegetation land coverage with NDVI >0.21, whereas in 2020 data show that most areas indicate low vegetation land coverage with NDVI <0.21. Data in 2019 show that VHI >33.67 which indicates that there is no drought, whereas in 2020 there is a decrease of areas with thick vegetated land coverage and an increase in areas indicating drought indicated by a VHI value <31.10. VCI values in the study area varied which VCI >50% shows a normal condition, VCI between 35%–50% shows a moderate level of drought and VCI <35% shows a high level of drought. Experiments using machine learning show that dBAI values in the study area are between −3.971 up to −2.376 which indicate that there is no land fire and there is new vegetation growth. DNBR values are between −0, 208 to −0.412 which indicate that vegetation starts to grow in the area. Prediction using machine learning shows that the ΔSVM and ΔCART algorithms produce the closest values to the ΔNBR observation data in 2018 and 2019. In future research, we will compare the performance of the framework between LANDSAT 8 OLI image data and Sentinel 2 image data. We will also compare the drought risk areas from vegetation indices analysis with meteorological methods such as SPI, SPEI and PDSI. The results of this comparison can be selected methodology that has high validation and accuracy in the software framework.

##  Supplemental Information

10.7717/peerj-cs.415/supp-1 Supplemental Information 1 Prediction result

10.7717/peerj-cs.415/supp-2 Supplemental Information 2 Machine Learning Data

10.7717/peerj-cs.415/supp-3 Supplemental Information 3 R Code of Machine Learning

10.7717/peerj-cs.415/supp-4 Supplemental Information 4 R Code Index vegetation Plot

## Additional Information and Declarations

### Data Availability

The following information was supplied regarding data availability: Data and code are available in the Supplementary Files.
